# A robust signature of immune‐related long non‐coding RNA to predict the prognosis of bladder cancer

**DOI:** 10.1002/cam4.4167

**Published:** 2021-08-10

**Authors:** Cong Lai, Zhenyu Wu, Zhuohang Li, Hao Yu, Kuiqing Li, Zhuang Tang, Cheng Liu, Kewei Xu

**Affiliations:** ^1^ Department of Urology Sun Yat‐sen Memorial Hospital Sun Yat‐sen University Guangzhou Guangdong P. R. China; ^2^ Guangdong Provincial Key Laboratory of Malignant Tumor Epigenetics and Gene Regulation Sun Yat‐sen Memorial Hospital Sun Yat‐sen University Guangzhou Guangdong P. R. China

**Keywords:** bladder cancer, immune, lncRNA, prognostic model, TCGA

## Abstract

**Background:**

Bladder cancer is the second most common malignant tumor in the urogenital system. The research investigated the prognostic role of immune‐related long non‐coding RNA (lncRNA) in bladder cancer.

**Methods:**

We extracted 411 bladder cancer samples from The Cancer Genome Atlas database. Single‐sample gene set enrichment analysis was employed to assess the immune cell infiltration of these samples. We recognized differentially expressed lncRNAs between tumors and paracancerous tissues, and differentially expressed lncRNAs between the high and low immune cell infiltration groups. Venn diagram analysis detected differentially expressed lncRNAs that intersected the above groups. LncRNAs with prognostic significance were identified by regression analysis. Multivariate Cox analysis was used to establish the risk score model. Then we established and evaluated the nomogram. Additionally, we performed gene set enrichment analysis to explore the potential functions of the screened lncRNAs in tumor pathogenesis.

**Results:**

Three hundred and twenty differentially expressed lncRNAs were recognized. We randomly divided patients into the training data set and the testing data set at a 2: 1 ratio. In the training data set, 9 immune‐related lncRNAs with prognostic significance were identified. The risk score model was constructed to classify patients as high‐ and low‐risk cohorts. Patients in the low‐risk cohort had better survival outcomes than those in the high‐risk cohort. The nomogram was established based on the indicators including age, gender, tumor‐node‐metastases stage, and risk score. The model's predictive performance was confirmed by the receiver operating characteristic curve analysis, concordance index method, calibration curve, and decision curve analysis. The testing data set also achieved similar results. Bioinformatics analysis suggested that the 9‐lncRNA signature was involved in the modulation of various immune responses, antigen processing and presentation, and T cell receptor signaling pathway.

**Conclusions:**

Our study uncovered the prognostic value of immune‐related lncRNAs for bladder cancer and showed that they may regulate tumor pathogenesis in various ways.

## Graphical abstract

The present study identified nine immune‐related long non‐coding RNA (lncRNA) and the 9‐lncRNAs signature possessed prognostic value for patients with bladder cancer. Bioinformatics analysis suggested that immune‐related lncRNAs may regulate tumor pathogenesis through the modulation of various immune responses, antigen processing and presentation, and T cell receptor signaling pathway.

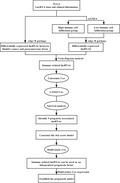

## 1 INTRODUCTION

Bladder cancer is the second most common urological malignancy in the world with approximately 573,000 new cases and 213,000 deaths in 2020.^1^ It was reported that the prognosis of bladder cancer patients was strictly related to the immune microenvironment of tumor tissues.^2^ Accumulated evidence has verified the therapeutic role of immune checkpoint inhibitors in bladder cancer, including atezolizumab, avelumab, durvalumab, nivolumab, and pembrolizumab.^3^ A recent research demonstrated that pembrolizumab could prolong the progression‐free survival of patients with high RNA‐based immune signature scores,^4^ which suggested that we might identify immune‐related prognostic indicators to improve the prognosis of bladder cancer patients and guide their treatment.

Long non‐coding RNAs (lncRNAs) are a group of RNAs that participate in the human physiological and pathological processes by interacting with specific RNAs and proteins. In recent years, it has been discovered that lncRNAs were involved in tumor growth and progression.^5^ In bladder cancer, lncRNA plays a vital role in lymphatic metastasis, epithelial‐mesenchymal transformation, proliferation, migration, and apoptosis of tumor cells.^6^, ^7^ LncRNA SOX2OT could maintain the stemness phenotype of bladder cancer stem cells and serve as an adverse indicator of clinical outcomes and prognosis.^8^ Furthermore, the exosomal lncRNA LNMAT2 could stimulate the formation and migration of lymphatic endothelial cells tube, and intensify the cancer lymphangiogenesis and lymphatic metastasis in bladder cancer.^9^ Therefore, lncRNA, as a novel biological marker, offers broad prospects for the early diagnosis and prognosis prediction of bladder cancer.

Studies have demonstrated that immune‐related lncRNAs have a unique value in the prognosis of several cancers. The heterogeneous expression of lncRNAs was identified among different immune‐infiltrating groups in muscle‐invasive bladder cancer.^10^ Shen et al.^11^ recognized 11 immune‐related lncRNAs as prognostic markers for breast cancer, whose signature was related to the infiltration of immune cell subtypes. Li et al.^12^ screened seven immune‐related lncRNAs in low‐grade glioma and confirmed that these lncRNAs have prognostic value in patients. Cao et al.^13^ have screened five immune‐related lncRNAs in bladder cancer but the signature's area under the curve (AUC) was relatively low (AUC = 0.666). Tong et al.^14^ have raised an epithelial‐mesenchymal transition‐related lncRNA signature in bladder cancer, which has included too many lncRNAs. Therefore, we aimed to propose a novel signature of immune‐related lncRNA to predict the prognosis of bladder cancer.

In the study, we analyzed the data set of lncRNAs and corresponding clinical information from the Cancer Genome Atlas (TCGA) and screened for immune‐related lncRNAs by single‐sample gene set enrichment analysis (ssGSEA). Furthermore, we established a prognostic model based on these lncRNAs and explored their potential biological functions in bladder cancer.

## 2 MATERIALS AND METHODS

### 2.1 Bladder cancer sample sources and grouping

Gene expression data (RNA‐Seq), lncRNA sequencing data, and corresponding clinical data of bladder cancer were downloaded from the TCGA database (https://portal.gdc.cancer.gov). Twenty‐nine immune cell data sets were applied to evaluate the infiltration level of immune cells through the ssGSEA method (Table S1).^15^ After that, patients were classified as the high and low immune cell infiltration groups using the hclust package. The stromal score, immune score, and tumor purity score were calculated by the ESTIMATE algorithm to verify the effectiveness of ssGSEA groupings.^16^ In addition, we assessed the difference between the two groups by analyzing the expression of the human leukocyte antigen (HLA) gene. CIBERSORT algorithm was employed to determine the infiltration of various immune cells in the tumor sample and verify the potency of the immune groupings again.^17^

### 2.2 Screening of immune‐related lncRNA

|log_2_Fold Change (FC)| > 0.5 and *p* < 0.05 were set as the standard to recognize the differentially expressed lncRNAs between the high and low immune cell infiltration groups by edgeR package. Differentially expressed lncRNAs between bladder cancer and adjacent tissues were also identified by the same method. Venn diagram analysis was used to screen out immune‐related lncRNAs in bladder cancer from the above two sets.

### 2.3 Construction of the risk score model

Patients with a follow‐up of more than 30 days were randomly divided into the training and testing sets at a ratio of 2:1 (cross‐validation method). In the training set, univariate Cox regression was performed on immune‐related lncRNAs and clinical data to identify prognosis‐related lncRNAs. We conducted the least absolute shrinkage and selection operator (LASSO) regression analysis to screen crucial lncRNAs which were tightly associated with overall survival (OS). Survival analyses were performed on the lncRNAs, respectively, to further screen lncRNAs with prognostic significance. The multivariate Cox regression model was utilized to calculate the respective coefficients (*β_i_*) of selected lncRNAs. Then, a risk score model consisting of *β_i_* and lncRNA expression levels (Expi) was established to appraise the risk score of each patient. We set the median risk score as a cutoff value and divided patients into high‐risk and low‐risk groups. Kaplan–Meier survival analysis was performed to compare the OS between the two groups. Receiver operating characteristic (ROC) curve analysis was utilized to evaluate the predictive efficacy of the model. The risk curve and scatter plot were generated to illustrate the risk score and survival status of each sample. The heatmap showed the expression profiles of the signature in the two groups. The correlation between risk scores and immune infiltration subtypes was analyzed by the Pearson correlation. The testing data set was applied to validate the above results.

### 2.4 Establishment and evaluation of the nomogram

We evaluated the prognostic significance of risk score and clinical variables such as age, gender, and tumor‐node‐metastases (TNM) stage by univariate and multivariate Cox regression analyses. The nomogram was established according to the results of multivariate Cox regression to predict each patient's 3‐ and 5‐year OS. We conducted the ROC curve analysis, concordance index (C‐index) method, calibration curve method, and decision curve analysis (DCA) to assess the model's accuracy. Finally, the testing set data were used to evaluate the above results.

### 2.5 Gene set enrichment analysis

We executed Gene Ontology (GO) enrichment analysis and Kyoto Encyclopedia of Genes and Genomes (KEGG) pathway analysis to investigate the potential pathways in which the immune‐related lncRNAs may participate.

### 2.6 Statistical analysis

All statistical analysis was accomplished by R version 3.6.2 (Institute for Statistics and Mathematics, Vienna, Austria; https://www.r‐project.org). The correlation was determined by Pearson correlation analysis. Chi‐square test and *t*‐test were utilized to compare clinical variables. Survival status was assessed by the Cox regression analysis. OS was generated by the Kaplan–Meier method and evaluated by the log‐rank test. Two‐tailed *p* < 0.05 was considered statistically significant.

## 3 RESULTS

### 3.1 Grouping and identification of bladder cancer samples

**FIGURE 1:**
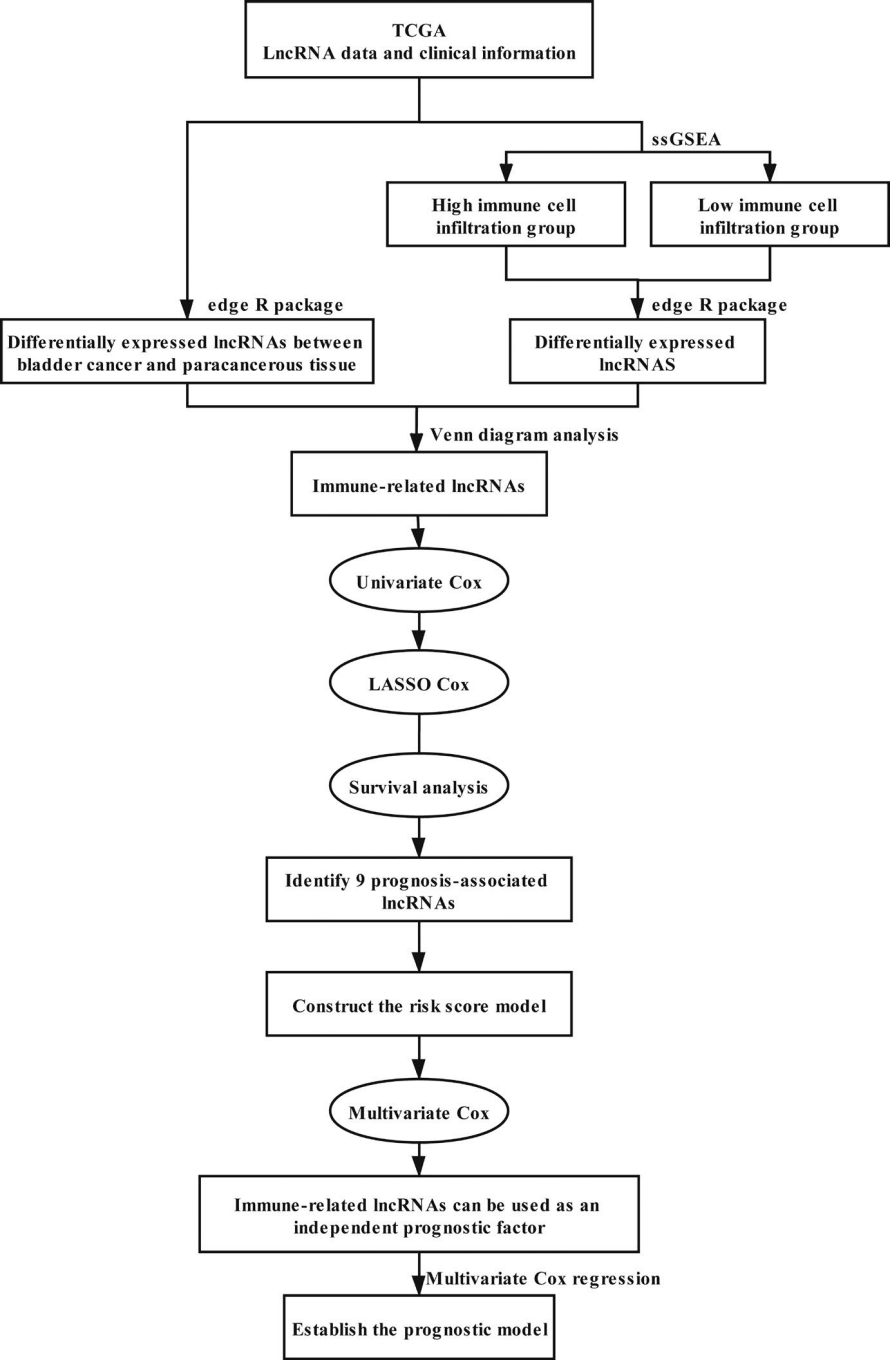
Flowchart for construction and evaluation of the prognostic model

**FIGURE 2:**
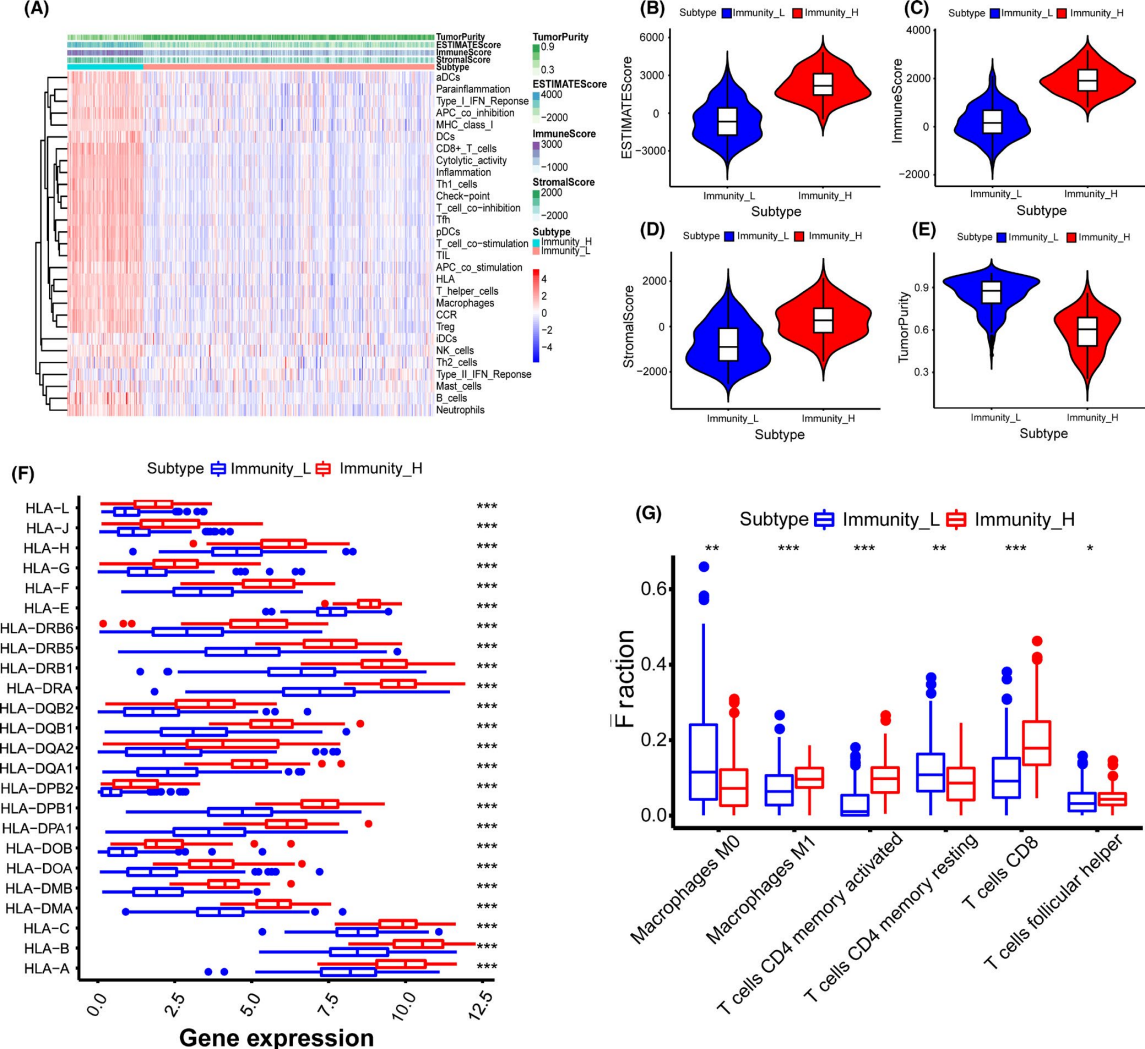
Establishment and verification of bladder cancer grouping. (A) The heatmap showed the unsupervised clustering of 29 immune cells in the high immune cell infiltration group (Immunity_H) and the low immune cell infiltration group (Immunity_L). Parameters including the tumor purity, ESTIMATE score, immune score, and stromal score were also displayed. (B–E) The box plots revealed the statistical differences in tumor purity, ESTIMATE score, immune score, and stromal score between the high and the low immune cell infiltration groups. (F) The expression of human leukocyte antigen (HLA) family genes in the high immune cell infiltration group was higher than that in the low immune cell infiltration group. (G) The CIBERSORT method demonstrated that a higher density of immune cells was found in the high immune cell infiltration group compared to the low immune cell infiltration group. *p < 0.05; **p < 0.01; ***p < 0.001

The flowchart of our research is shown in Figure 1. Information on 411 bladder cancer tissues and 19 adjacent tissues were obtained from the TCGA database. Three hundred and ninety‐three patients with a follow‐up of more than 30 days were included in the study (Table S2). The transcriptome data of bladder cancer samples were analyzed by the ssGSEA method to assess the level of immune cell infiltration level. An unsupervised hierarchical clustering algorithm was employed to divide patients into the high immune cell infiltration group (*n* = 85) and the low immune cell infiltration group (*n* = 326) (Figure 2A). The ESTIMATE algorithm was used to calculate the ESTIMATE score, immune score, stromal score, and tumor purity of all samples. Compared with the low immune cell infiltration group, the high immune cell infiltration group presented higher ESTIMATE score, higher immune score, higher stromal score, and lower tumor purity (*p* < 0.001) (Figure 2B–E). The expression of HLA family genes in the high immune cell infiltration group was higher than that in the low immune cell infiltration group (*p* < 0.001) (Figure 2F). In addition, the CIBERSORT method revealed that the high immune cell infiltration group had a higher density of immune cells (Figure 2G). Overall, our results indicated that the bladder cancer grouping was feasible.

### 3.2 Identification of the immune‐related lncRNAs

**FIGURE 3:**
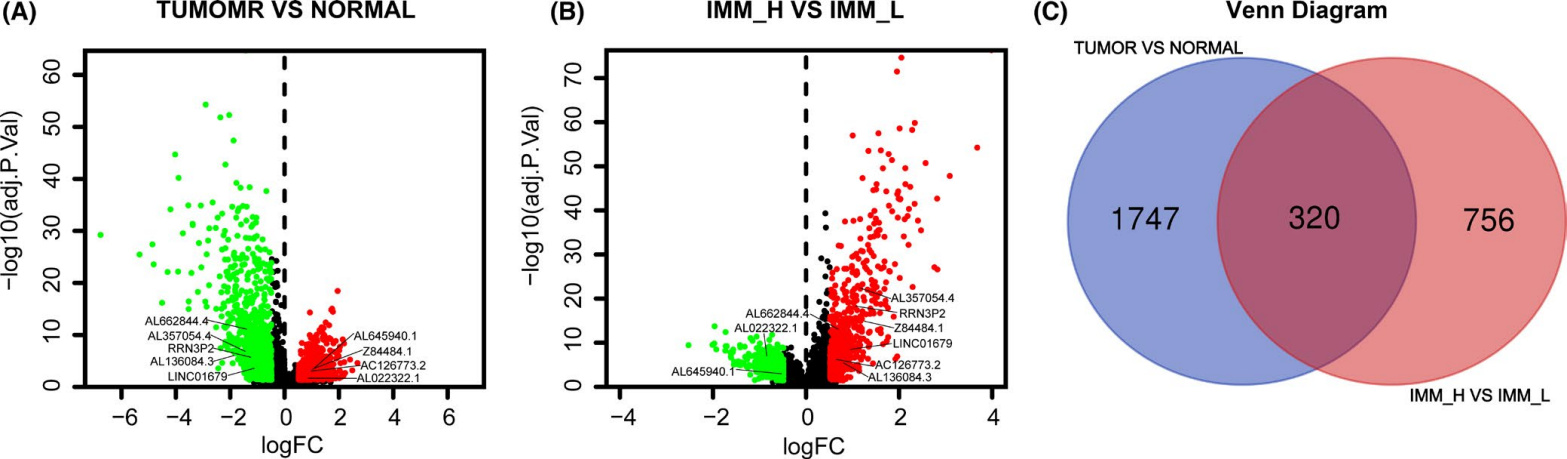
Identification of immune‐related long non‐coding RNA (lncRNA) in bladder cancer. (A) The volcano plot visualized that 1178 lncRNAs were up‐regulated and 889 were down‐regulated in bladder cancer compared to paracancerous tissues. (B) The volcano plot indicated that 440 immune‐related genes were up‐regulated and 636 were down‐regulated in the high immune cell infiltration group compared to the low immune cell infiltration group. (C) The Venn diagram analysis detected 320 differentially expressed lncRNAs that intersected the above groups

We recognized 2067 differentially expressed lncRNAs between tumors and adjacent tissues (Figure 3A) and 1076 differentially expressed lncRNAs between the high and low immune cell infiltration groups (Figure 3B). The Venn diagram analysis detected 320 differentially expressed lncRNAs that intersected the above groups (Figure 3C). Taking together, we screened 320 immune‐related lncRNAs in bladder cancer.

### 3.3 Construction and assessment of the risk score model

**FIGURE 4:**
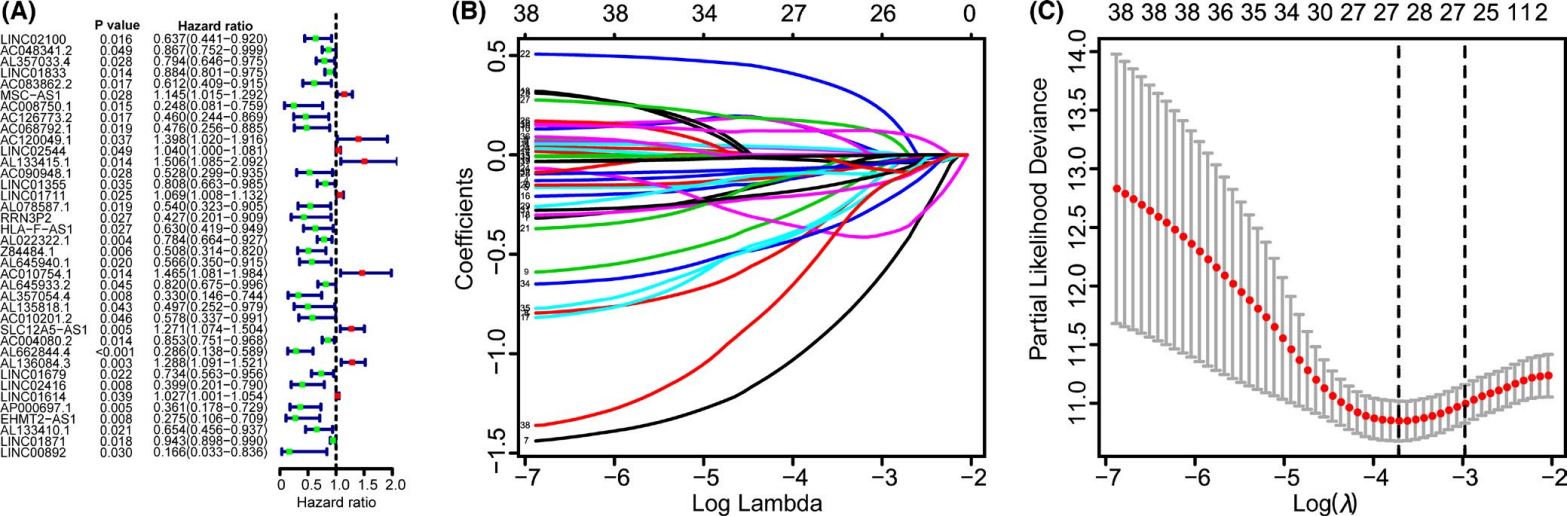
Identification of immune‐related long non‐coding RNAs (lncRNAs) with prognostic significance. (A) The risk ratio forest plot visualized 38 immune‐related lncRNAs significantly associated with the overall survival. (B) The least absolute shrinkage and selection operator (LASSO) analysis identified 28 lncRNAs tightly correlated with prognosis. (C) Illustration of LASSO coefficient spectrum for prognosis‐related lncRNAs

**FIGURE 5:**
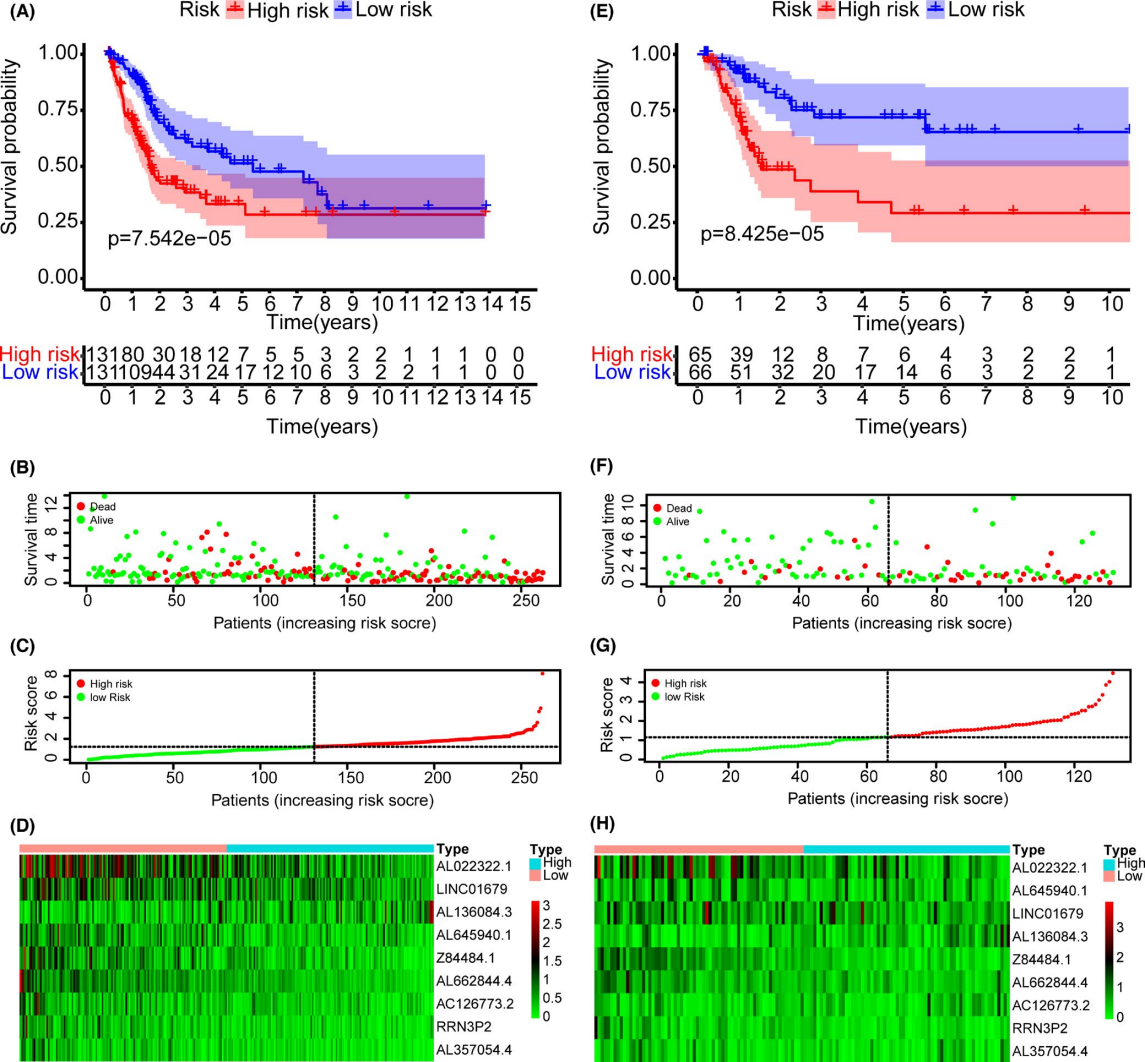
Construction of the risk‐score model based on immune‐related long non‐coding RNAs (lncRNAs). (A, E) Kaplan–Meier analysis showed that patients in the high‐risk group suffered worse overall survival compared to the low‐risk group in training and testing data sets, respectively. (B, F) The overviews of survival time for each patient in training and testing data sets, respectively. (C, G) The distributions of a risk score for each patient in training and testing data sets, respectively. (D, H) The heatmaps of expression profiles for 9‐lncRNAs signature between the low‐risk and high‐risk groups in training and testing data sets, respectively. Warm colors represented high expression, while cold colors represented low expression

In the training data set, univariate Cox regression was performed on immune‐related lncRNAs to identify 38 prognosis‐associated lncRNAs (Figure 4A). LASSO regression analysis further screened nine crucial lncRNAs (Figure 4B,C). Survival analyses of immune‐related lncRNAs revealed that nine lncRNAs were significantly related to OS, including AC126773.2 (*p* = 0.039), RRN3P2 (*p* = 0.04), AL022322.1 (*p* = 0.003), Z84484.1 (*p* = 0.00001), AL645940.1 (*p* = 0.008), AL357054.4 (*p* = 0.043), AL662844.4 (*p* = 0.04), AL136084.3 (*p* = 0.013), and LINC01679 (*p* = 0.035) (Figure S1). *β_i_* was calculated (Table 1) to establish the risk score model: Risk score = $\sum_{i=1}^{9}\left(\beta_{i}*\exp_{i}\right)$. We set the median risk score as a cutoff value and divided 411 patients into high‐risk and low‐risk groups. The Kaplan–Meier curve disclosed that the OS in the low‐risk group was significantly better than that in the high‐risk group (*p* = 7.542e–05) (Figure 5A). The risk curve and scatter plot indicated that the risk coefficient and mortality of patients in the high‐risk group were higher than those in the low‐risk group (Figure 5B,C). The heatmap exhibited the expression profiles of the 9‐lncRNAs signature in the two groups (Figure 5D). The correlation status of B cells, CD4^+^ T cells, CD8^+^ T cells, dendritic cells, macrophages, and neutrophils with risk score were plotted in Figure S2. The correlation values (infiltration status) of B cells, CD4^+^ T cells, CD8^+^ T cells, dendritic cells, macrophages, and neutrophils with risk score were −0.157, −0.200, 0.167, −0.005, 0.428, and −0.046, respectively, in the training data set. And the correlation values of B cells, CD4^+^ T cells, CD8^+^ T cells, dendritic cells, macrophages, and neutrophils with risk score were −0.186, −0.009, 0.106, −0.061, 0.239, and 0.036, respectively, in the testing data set. Similar results were obtained using the same method on the testing data set (Figure 5E–H).

**TABLE 1:** The prognostic significance of the 9‐long non‐coding RNAs signature

Immune‐related gene	Coef	Hazard ratio (HR)
AC126773.2	−0.245674785	0.782176559
RRN3P2	−0.439240487	0.644525761
AL022322.1	−0.210005673	0.810579648
Z84484.1	−0.262440116	0.769172424
AL645940.1	−0.136995376	0.871974258
AL357054.4	−0.717602304	0.48792074
AL662844.4	−0.731805115	0.481039873
AL136084.3	0.182874542	1.200663765
LINC01679	−0.174892219	0.839547503

### 3.4 Construction and evaluation of the prognostic model

**FIGURE 6:**
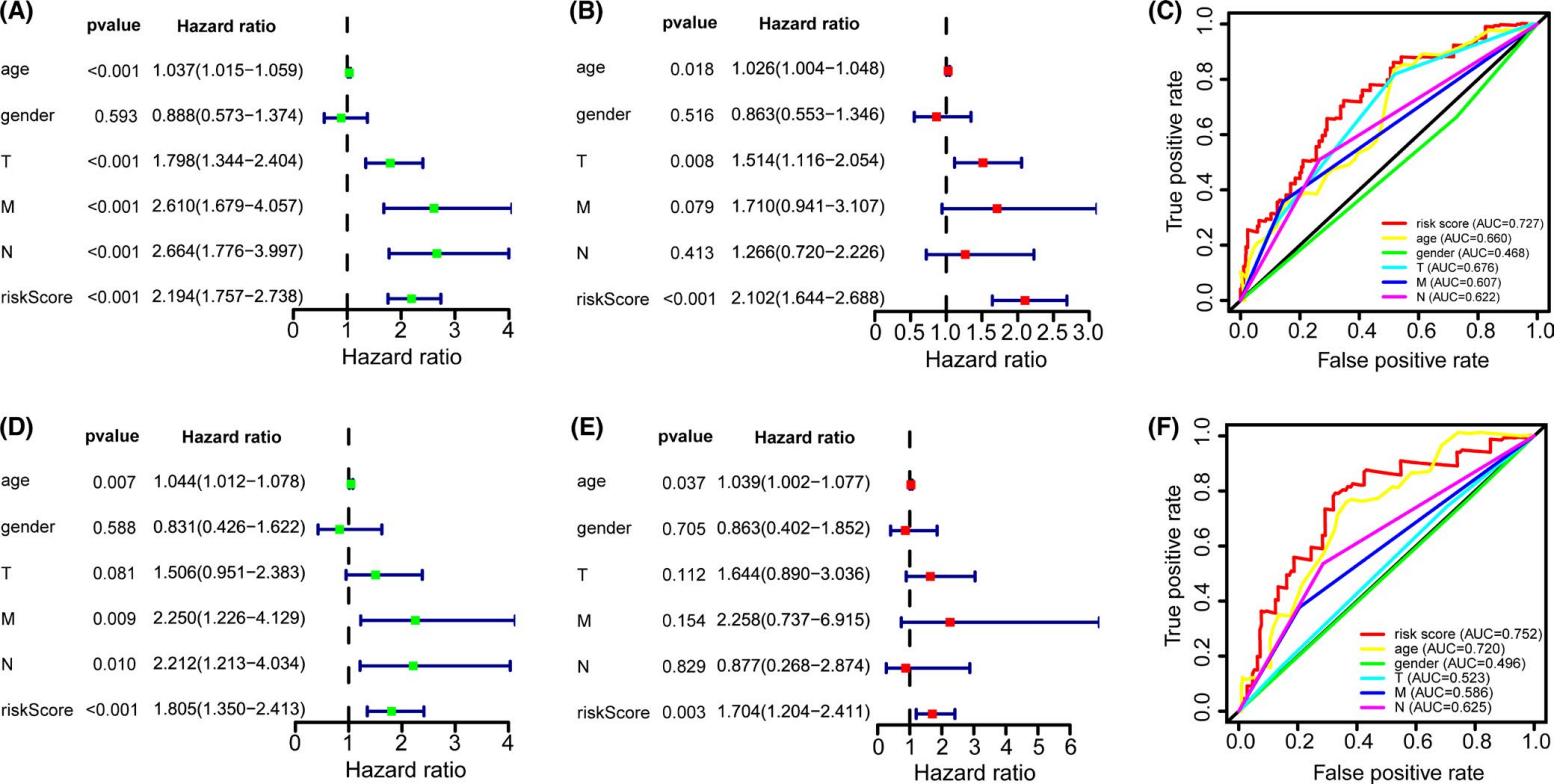
The prognostic value of risk score and clinical variables. (A, D) Univariate Cox analysis showed that risk score and clinical variables including age, gender, and TNM stage were significantly related to overall survival (OS) in training and testing data sets, respectively. (B, E) Multivariate Cox analysis manifested that the 9‐long non‐coding RNAs (lncRNAs) signature was an independent prognostic indicator for bladder cancer in training and testing data sets, respectively. (C, F) ROC curve analysis of the 9‐lncRNAs signature demonstrated that area under the curves in the training data set and in the testing data set were 0.727 and 0.752, respectively

**FIGURE 7:**
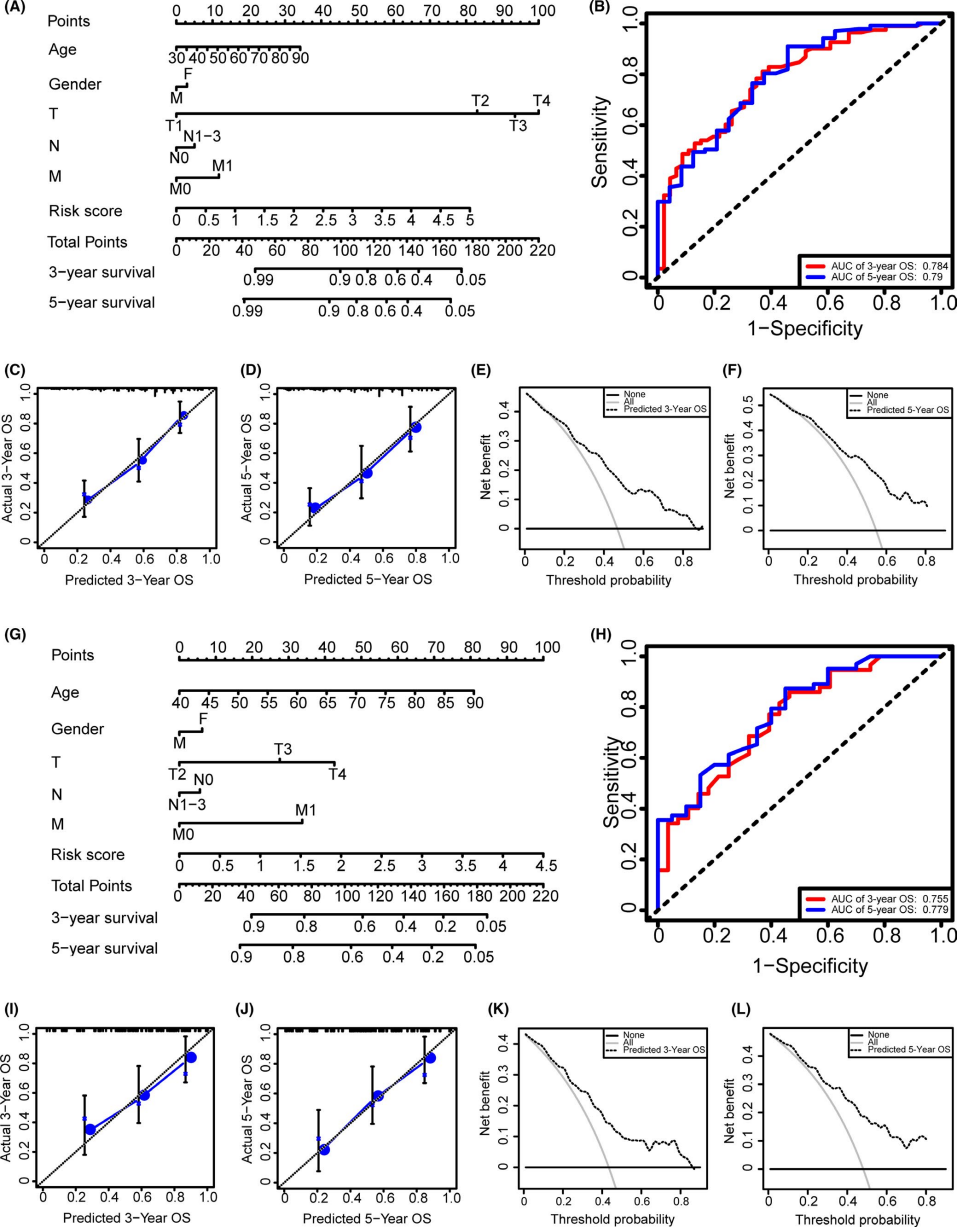
Establishment and evaluation of the prognostic model. (A, G) The nomograms for predicting the patients' overall survival (OS) in training and testing data sets, respectively. (B, H) ROC curve analysis showed that area under the curves (AUCs) of 3‐ and 5‐year OS were 0.784 and 0.790, respectively, in the training data set, and AUCs of 3‐ and 5‐year OS were 0.755 and 0.779, respectively, in the testing data set. (C, I) The calibration curves for 3‐year OS of the nomogram in training and testing data sets, respectively. (D, J) The calibration curves for 5‐year OS of the nomogram in training and testing data sets, respectively. (E, K) The DCA for 3‐year OS of the nomogram in training and testing data sets, respectively. (F, L) The DCA of 5‐year OS of the nomogram in training and testing data sets, respectively

Univariate Cox regression showed that the risk score and clinical indicators including age, gender, and TNM stage were firmly related to OS (Figure 6A). We further conducted the multivariate Cox analysis and found that the 9‐lncRNAs signature was an independent prognostic factor for bladder cancer (*p* < 0.001) (Figure 6B). ROC curve analysis validated the predictive performance of the signature (Figure 6C). We then established a nomogram including age, gender, TNM stage, and risk score (Figure 7A). The AUCs for 3‐, 5‐year OS predicted by the model were 0.784 and 0.790, respectively (Figure 7B). The C‐index of the nomogram was 0.751. The calibration curves and DCAs of the prognostic model showed that the model had an excellent predictive effect (Figure 7C–F). We acquired similar results using the same method on the testing data set (Figures 6D–F and 7G–L).

### 3.5 Gene set enrichment analysis

**FIGURE 8:**
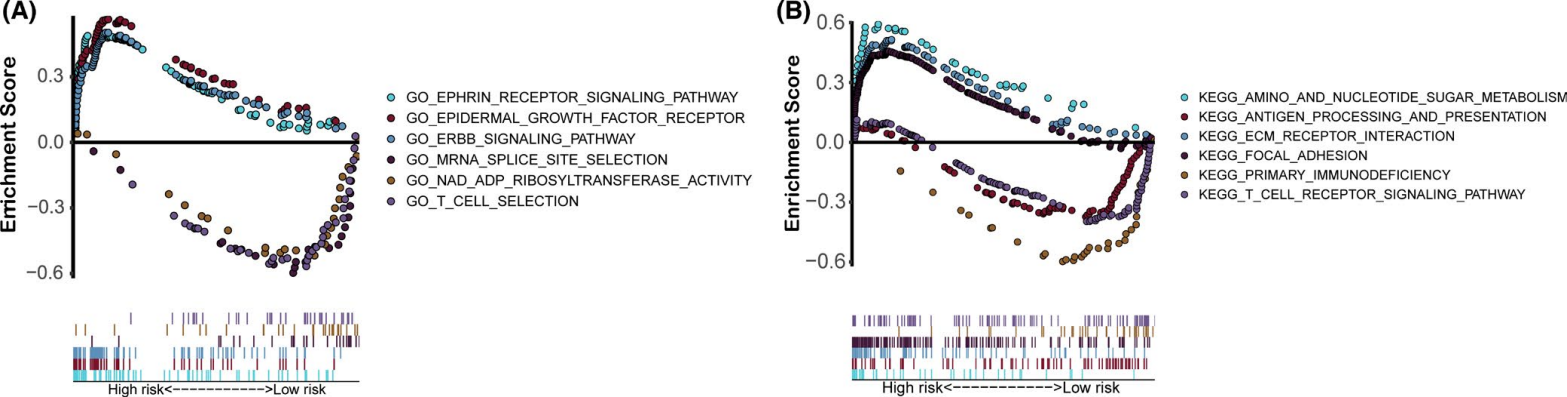
Gene set enrichment analysis on the differentially expressed genes between the high‐risk and low‐risk groups. (A) Gene Ontology (GO) enrichment analysis indicated that the genes were enriched in the ephrin receptor signaling pathway, epidermal growth factor receptor signaling pathway, ERBB signaling pathway, mRNA splice site selection, DNA ADP ribosyltransferase activity, and T cell selection. (B) Kyoto Encyclopedia of Genes and Genomes (KEGG) pathway analysis showed that these genes were involved in amino sugar and nucleotide sugar metabolism, antigen processing and presentation, extracellular matrix receptor interaction, focal adhesion, primary immunodeficiency, and T cell receptor signaling pathway

We performed GO enrichment analysis and KEGG pathway analysis on the differentially expressed genes between the high‐risk and low‐risk groups. GO enrichment analysis indicated that the genes were enriched in the ephrin receptor signaling pathway, epidermal growth factor receptor (EGFR) signaling pathway, ERBB signaling pathway, mRNA splicing site selection, DNA adenosine diphosphate (ADP) ribosyltransferase activity, and T cell selection (Figure 8A). KEGG pathway analysis showed that these genes were involved in amino sugar and nucleotide sugar metabolism, antigen processing and presentation, extracellular matrix (ECM) receptor interaction, focal adhesion, primary immunodeficiency, and T cell receptor signaling pathway (Figure 8B).

## 4 DISCUSSION

Bladder cancer has the characteristics of a high recurrence rate and poor prognosis.^18^ Accurately predicting the prognosis of bladder cancer patients is of great importance for guiding their treatment. Prognostic models based on immune‐related genes have been developed and proved to have excellent predictive efficacy in bladder cancer patients.^19^, ^20^ Using lncRNAs to construct a prognostic model may be an important supplement to predict the prognosis of bladder cancer.

Accumulated studies have shown that lncRNA plays an essential role in the tumor immune microenvironment. LncRNA NKILA activates T cell‐induced cell death to promote tumor immune escape.^21^ LncRNA SNHG1 regulates the differentiation of Treg cells by targeting miR‐448, thereby affecting the immune escape of breast cancer.^22^ The above researches indicated that these lncRNAs might have prognostic value in cancer patients. Furthermore, Zhou et al.^23^ identified six immune‐related lncRNAs in glioblastoma and confirmed that these lncRNAs had prognostic value in glioblastoma patients. Therefore, we proposed a novel signature of immune‐related lncRNA to predict the prognosis of bladder cancer.

We analyzed the lncRNAs data set from the TCGA database and screened 320 immune‐related lncRNAs. Nine immune‐related lncRNAs with prognostic significance were ultimately identified. Furthermore, our findings indicated that AL136084.3 was an adverse prognostic factor for bladder cancer, whereas the other lncRNAs were favorable prognostic factors. Multivariate Cox analysis was used to construct the risk score model. We found that patients in the low‐risk group had longer OS than that of the high‐risk group. In addition, we noticed that the infiltration of B cells was significantly negatively correlated with the prognosis of bladder cancer, while that of macrophages was on the contrary. B cells have long been recognized as effector cells of humoral immunity that inhibited tumor progression through secreting immunoglobulins, activating T cells, and killing tumor cells directly.^24^ A high density of CD20^+^ B cells was independently correlated with a prolonged time to recurrence in bladder cancer.^25^ Moreover, macrophages could contribute to tumor progression by accelerating genetic instability, promoting metastasis, nurturing cancer stem cells, and taming protective immunity.^26^ In bladder cancer, a higher expression of M2 macrophage is associated with a worse grade and stage of the tumor.^27^

Subsequently, we established a nomogram including age, gender, TNM stage, and risk score. The ROC curve analysis, C‐index, calibration curves, and DCA confirmed the model's predictive power. Compared to models based on other sequencing data, the prognostic model constructed by immune‐related lncRNAs presented better efficacy according to the ROC curve method.^28^, ^29^, ^30^, ^31^

Furthermore, we performed GO enrichment analysis and KEGG pathway analysis to explore the potential functions of the 9‐lncRNAs signature in bladder cancer. The results showed that these lncRNAs were involved in various immune responses, antigen processing, and presentation, T cell receptor signaling pathway, EGFR signaling pathway, ERBB signaling pathway, ECM receptor interaction, focal adhesion, and primary immunodeficiency. Epidermal growth factor was reported to activate the androgen receptor and increase the expression of TRIP13 to promote bladder cancer progression.^32^, ^33^ Notably, ECM modification could not only promote tumor cells to escape, but also help generate and maintain the cancer stem cell niche.^34^ Moreover, high infiltration of memory‐activated CD4^+^ T cell subsets were associated with prolonged OS and reduced risk of tumor recurrence in bladder cancer.^35^ Chobrutskiy et al.^36^ demonstrated that lower CDR3 region isoelectric point in T cell receptor was associated with better survival outcomes in bladder cancer.

However, there are some limitations to our research. It is a retrospective study whose data were obtained from the TCGA database that lacked information including treatment and recurrence records. In vivo or in vitro experiments and prospective clinical researches are needed to validate our conclusions.

## 5 CONCLUSION

In summary, the present study identified a 9‐lncRNAs signature that possessed prognostic value for bladder cancer patients. The immune‐related lncRNAs may regulate tumor pathogenesis through the modulation of various immune responses, antigen processing and presentation, and T cell receptor signaling pathway. Our research proposes a predictive model and biomarkers for bladder cancer patients.

## CONFLICT OF INTEREST

The authors declare no conflicts of interest.

## AUTHOR CONTRIBUTION

Kewei Xu and Cheng Liu designed and directed the research. Cong Lai, Zhenyu Wu, and Zhuohang Li analyzed the data and drafted the manuscript. Hao Yu and Kuiqing Li collected references and revised the manuscript. Zhuang Tang collected the data. All authors read and approved the final manuscript.

## Supporting information

Fig S1

Fig S2

Table S1

Table S2

Supplementary Material

## Data Availability

The original contributions presented in the study are publicly available. This data can be found here: https://portal.gdc.cancer.gov (TCGA‐BLCA).

## References

[1] Sung H, Ferlay J, Siegel RL, et al. Global cancer statistics 2020: GLOBOCAN estimates of incidence and mortality worldwide for 36 cancers in 185 countries. CA Cancer J Clin. 2021;71(3):209‐249. 33538338 10.3322/caac.21660

[2] Song D, Powles T, Shi L, Zhang L, Ingersoll MA, Lu YJ. Bladder cancer, a unique model to understand cancer immunity and develop immunotherapy approaches. J Pathol. 2019;249(2):151‐165. 31102277 10.1002/path.5306 PMC6790662

[3] Grimm MO, Bex A, De Santis M, et al. Safe use of immune checkpoint inhibitors in the multidisciplinary management of urological cancer: the European Association of Urology Position in 2019. Eur Urol. 2019;76(3):368‐380. 31235192 10.1016/j.eururo.2019.05.041

[4] Necchi A, Raggi D, Gallina A, et al. Impact of molecular subtyping and immune infiltration on pathological response and outcome following neoadjuvant pembrolizumab in muscle‐invasive bladder cancer. Eur Urol. 2020;77(6):701‐710. 32165065 10.1016/j.eururo.2020.02.028

[5] Huang Z, Zhou JK, Peng Y, He W, Huang C. The role of long noncoding RNAs in hepatocellular carcinoma. Mol Cancer. 2020;19(1):77. 32295598 10.1186/s12943-020-01188-4 PMC7161154

[6] Martens‐Uzunova ES, Böttcher R, Croce CM, Jenster G, Visakorpi T, Calin GA. Long noncoding RNA in prostate, bladder, and kidney cancer. Eur Urol. 2014;65(6):1140‐1151. 24373479 10.1016/j.eururo.2013.12.003

[7] Zhang Q, Su M, Lu G, Wang J. The complexity of bladder cancer: long noncoding RNAs are on the stage. Mol Cancer. 2013;12(1):101. 24006935 10.1186/1476-4598-12-101 PMC3846905

[8] Zhan Y, Chen Z, He S, et al. Long non‐coding RNA SOX2OT promotes the stemness phenotype of bladder cancer cells by modulating SOX2. Mol Cancer. 2020;19(1):25. 32019566 10.1186/s12943-020-1143-7 PMC6998848

[9] Chen C, Luo Y, He W, et al. Exosomal long noncoding RNA LNMAT2 promotes lymphatic metastasis in bladder cancer. J Clin Invest. 2020;130(1):404‐421. 31593555 10.1172/JCI130892 PMC6934220

[10] Jiang W, Zhu D, Wang C, Zhu Y. An immune relevant signature for predicting prognoses and immunotherapeutic responses in patients with muscle‐invasive bladder cancer (MIBC). Cancer Med. 2020;9(8):2774‐2790. 32096345 10.1002/cam4.2942 PMC7163112

[11] Shen Y, Peng X, Shen C. Identification and validation of immune‐related lncRNA prognostic signature for breast cancer. Genomics. 2020;112(3):2640‐2646. 32087243 10.1016/j.ygeno.2020.02.015

[12] Li X, Meng Y. Survival analysis of immune‐related lncRNA in low‐grade glioma. BMC Cancer. 2019;19(1):813. 31419958 10.1186/s12885-019-6032-3 PMC6697914

[13] Cao R, Yuan L, Ma B, Wang G, Tian Y. Immune‐related long non‐coding RNA signature identified prognosis and immunotherapeutic efficiency in bladder cancer (BLCA). Cancer Cell Int. 2020;20:276. 32607061 10.1186/s12935-020-01362-0 PMC7320553

[14] Tong H, Li T, Gao S, Yin H, Cao H, He W. An epithelial‐mesenchymal transition‐related long noncoding RNA signature correlates with the prognosis and progression in patients with bladder cancer. Biosci Rep. 2021;41(1):BSR20203944. 33289830 10.1042/BSR20203944 PMC7786330

[15] Bindea G, Mlecnik B, Tosolini M, et al. Spatiotemporal dynamics of intratumoral immune cells reveal the immune landscape in human cancer. Immunity. 2013;39(4):782‐795. 24138885 10.1016/j.immuni.2013.10.003

[16] Yoshihara K, Shahmoradgoli M, Martínez E, et al. Inferring tumour purity and stromal and immune cell admixture from expression data. Nat Commun. 2013;4:2612. 24113773 10.1038/ncomms3612 PMC3826632

[17] Newman AM, Liu CL, Green MR, et al. Robust enumeration of cell subsets from tissue expression profiles. Nat Methods. 2015;12(5):453‐457. 25822800 10.1038/nmeth.3337 PMC4739640

[18] Bray F, Ferlay J, Soerjomataram I, Siegel RL, Torre LA, Jemal A. Global cancer statistics 2018: GLOBOCAN estimates of incidence and mortality worldwide for 36 cancers in 185 countries. CA Cancer J Clin. 2018;68(6):394‐424. 30207593 10.3322/caac.21492

[19] Qiu H, Hu X, He C, Yu B, Li Y, Li J. Identification and validation of an individualized prognostic signature of bladder cancer based on seven immune related genes. Front Genet. 2020;11:12. 32117435 10.3389/fgene.2020.00012 PMC7013035

[20] Cao J, Yang X, Li J, et al. Screening and identifying immune‐related cells and genes in the tumor microenvironment of bladder urothelial carcinoma: based on TCGA database and bioinformatics. Front Oncol. 2019;9:1533. 32010623 10.3389/fonc.2019.01533 PMC6974676

[21] Huang D, Chen J, Yang L, et al. NKILA lncRNA promotes tumor immune evasion by sensitizing T cells to activation‐induced cell death. Nat Immunol. 2018;19(10):1112‐1125. 30224822 10.1038/s41590-018-0207-y

[22] Pei X, Wang X, Li H. LncRNA SNHG1 regulates the differentiation of Treg cells and affects the immune escape of breast cancer via regulating miR‐448/IDO. Int J Biol Macromol. 2018;118(Pt A):24‐30. 29886172 10.1016/j.ijbiomac.2018.06.033

[23] Zhou M, Zhang Z, Zhao H, Bao S, Cheng L, Sun J. An immune‐related six‐lncRNA signature to improve prognosis prediction of glioblastoma multiforme. Mol Neurobiol. 2018;55(5):3684‐3697. 28527107 10.1007/s12035-017-0572-9

[24] Tokunaga R, Naseem M, Lo JH, et al. B cell and B cell‐related pathways for novel cancer treatments. Cancer Treat Rev. 2019;73:10‐19. 30551036 10.1016/j.ctrv.2018.12.001 PMC7505165

[25] Wahlin S, Nodin B, Leandersson K, Boman K, Jirström K. Clinical impact of T cells, B cells and the PD‐1/PD‐L1 pathway in muscle invasive bladder cancer: a comparative study of transurethral resection and cystectomy specimens. Oncoimmunology. 2019;8(11):e1644108. 31646091 10.1080/2162402X.2019.1644108 PMC6791444

[26] Mantovani A, Marchesi F, Malesci A, Laghi L, Allavena P. Tumour‐associated macrophages as treatment targets in oncology. Nat Rev Clin Oncol. 2017;14(7):399‐416. 28117416 10.1038/nrclinonc.2016.217 PMC5480600

[27] Sharifi L, Nowroozi MR, Amini E, Arami MK, Ayati M, Mohsenzadegan M. A review on the role of M2 macrophages in bladder cancer; pathophysiology and targeting. Int Immunopharmacol. 2019;76: 105880. 31522016 10.1016/j.intimp.2019.105880

[28] He A, He S, Peng D, et al. Prognostic value of long non‐coding RNA signatures in bladder cancer. Aging (Albany NY). 2019;11(16):6237‐6251. 31433789 10.18632/aging.102185 PMC6738399

[29] Zhao D, Peng Q, Wang L, et al. Identification of a six‐lncRNA signature based on a competing endogenous RNA network for predicting the risk of tumour recurrence in bladder cancer patients. J Cancer. 2020;11(1):108‐120. 31892978 10.7150/jca.35801 PMC6930402

[30] Yin XH, Jin YH, Cao Y, et al. Development of a 21‐miRNA signature associated with the prognosis of patients with bladder cancer. Front Oncol. 2019;9:729. 31448232 10.3389/fonc.2019.00729 PMC6692470

[31] Wang L, Shi J, Huang Y, et al. A six‐gene prognostic model predicts overall survival in bladder cancer patients. Cancer Cell Int. 2019;19:229. 31516386 10.1186/s12935-019-0950-7 PMC6729005

[32] Izumi K, Zheng Y, Li Y, Zaengle J, Miyamoto H. Epidermal growth factor induces bladder cancer cell proliferation through activation of the androgen receptor. Int J Oncol. 2012;41(5):1587‐1592. 22922989 10.3892/ijo.2012.1593 PMC3583640

[33] Gao Y, Liu S, Guo Q, et al. Increased expression of TRIP13 drives the tumorigenesis of bladder cancer in association with the EGFR signaling pathway. Int J Biol Sci. 2019;15(7):1488‐1499. 31337978 10.7150/ijbs.32718 PMC6643140

[34] Malanchi I, Santamaria‐Martínez A, Susanto E, et al. Interactions between cancer stem cells and their niche govern metastatic colonization. Nature. 2011;481(7379):85‐89. 22158103 10.1038/nature10694

[35] Zhang C, Shen L, Qi F, Wang J, Luo J. Multi‐omics analysis of tumor mutation burden combined with immune infiltrates in bladder urothelial carcinoma. J Cell Physiol. 2020;235(4):3849‐3863. 31596511 10.1002/jcp.29279 PMC13482692

[36] Chobrutskiy BI, Zaman S, Diviney A, Mihyu MM, Blanck G. T‐cell receptor‐α CDR3 domain chemical features correlate with survival rates in bladder cancer. J Cancer Res Clin Oncol. 2019;145(3):615‐623. 30539280 10.1007/s00432-018-2815-1 PMC11810338

