# Supplementary figures and images for: A robust signature of immune‐related long non‐coding RNA to predict the prognosis of bladder cancer
Source: Cancer Med. 2021 Aug 10;10(18):6534–45. doi: 10.1002/cam4.4167 (PMC8446409; doi:10.1002/cam4.4167)

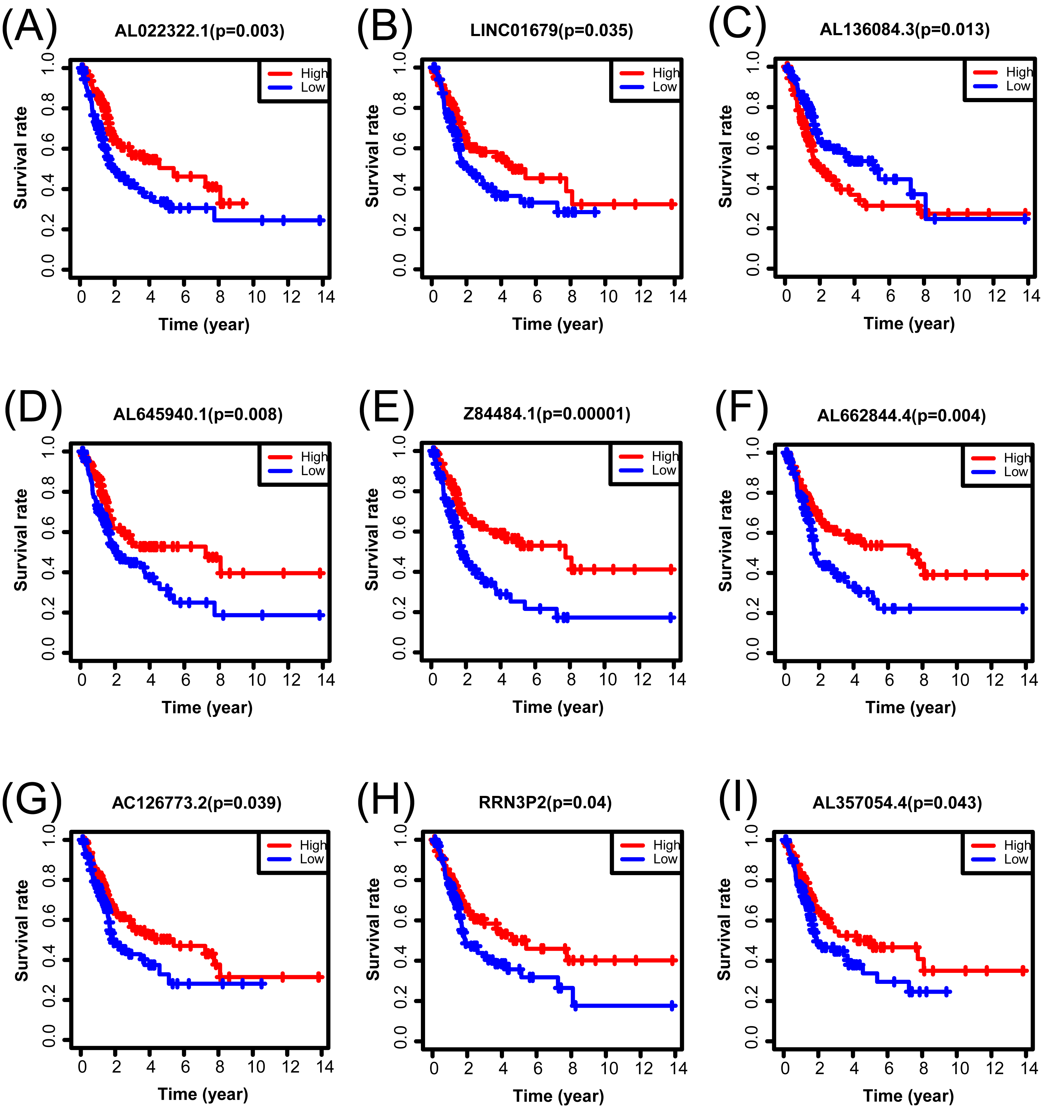

Supplement: Supplementary file 1 — Fig S1 [file CAM4-10-6534-s002.tif]

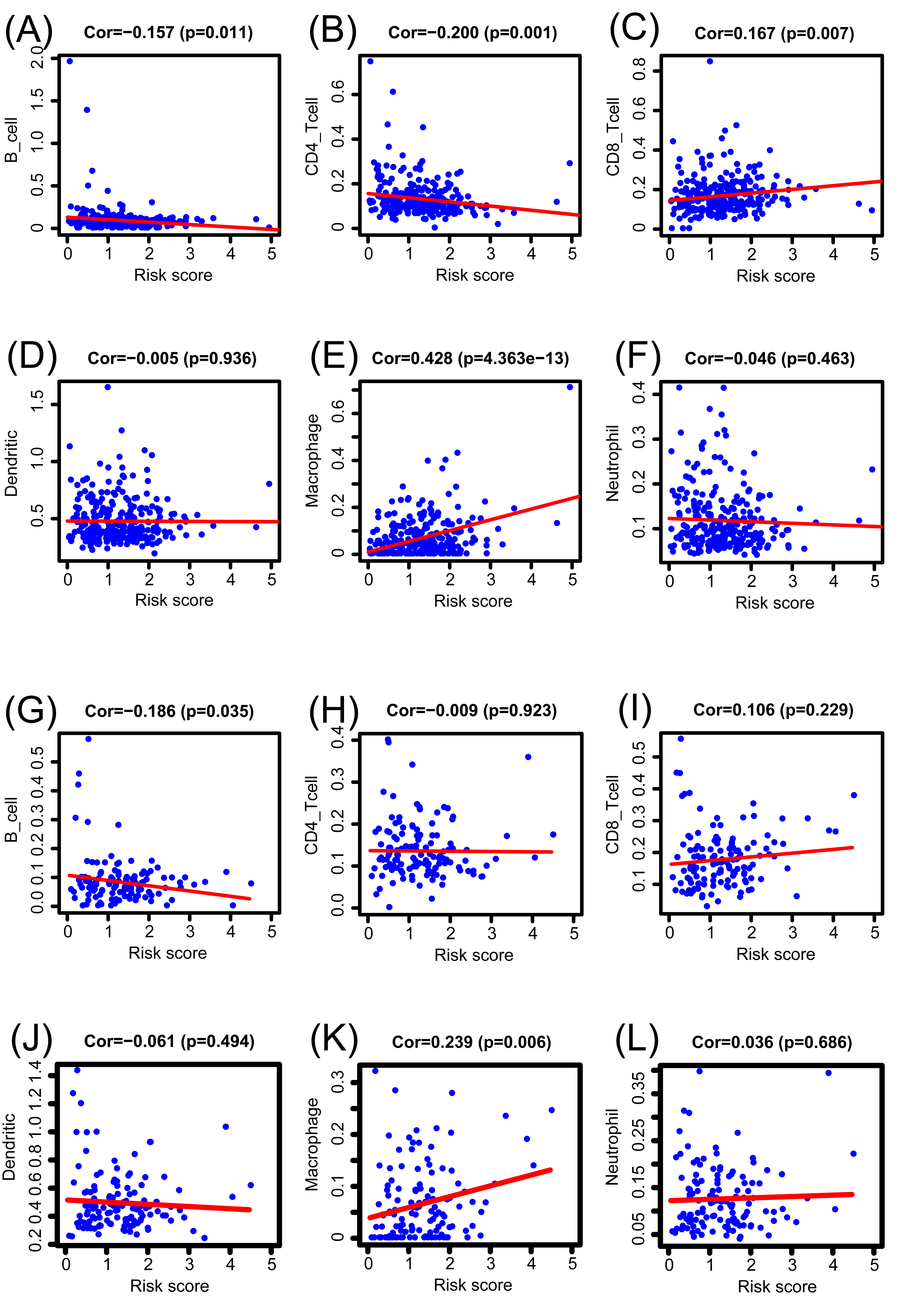

Supplement: Supplementary file 2 — Fig S2 [file CAM4-10-6534-s004.tif]
