# Supplementary material for: A robust signature of immune‐related long non‐coding RNA to predict the prognosis of bladder cancer
Source: Cancer Med. 2021 Aug 10;10(18):6534–45. doi: 10.1002/cam4.4167 (PMC8446409; doi:10.1002/cam4.4167)
Supplement: Supplementary file 4 — Table S2 [file CAM4-10-6534-s001.docx]

**Supplementary Table 2. Clinical characteristics of all included 393 patients from TCGA in the research**

|  | Training data set | Testing data set |
| --- | --- | --- |
| Sample size | 262 | 131 |
| Follow-up (years) | 2.08 ± 2.22 | 2.23 ± 2.26 |
| Vital status |  |  |
| Alive | 154 (58.8) | 87 (66.4) |
| Dead | 108 (41.2) | 44 (33.6) |
| Age | 67.15 ± 10.41 | 66.94 ± 10.35 |
| Gender |  |  |
| Female | 55 (21.0) | 30 (22.9) |
| Male | 184 (70.2) | 98 (74.8) |
| NA | 23 (8.8) | 3 (2.3) |
| T stage |  |  |
| T2 | 91 (34.7) | 47 (35.9) |
| T3 | 112 (42.7) | 63 (48.1) |
| T4 | 36 (13.8) | 18 (13.7) |
| NA | 23 (8.8) | 3 (2.3) |
| N stage |  |  |
| N0 | 164 (62.6) | 90 (68.7) |
| N1-3 | 75 (28.6) | 38 (29.0) |
| NA | 23 (8.8) | 3 (2.3) |
| M stage |  |  |
| M0 | 199 (76.0) | 120 (91.6) |
| M1 | 40 (15.2) | 8 (6.1) |
| NA | 23 (8.8) | 3 (2.3) |
| Risk score | 1.30 ± 0.86 | 1.26 ± 0.82 |
| Risk status |  |  |
| High | 131 (50.0) | 65 (49.6) |
| Low | 131 (50.0) | 66 (50.4) |
