# Supplementary material for: A robust signature of immune‐related long non‐coding RNA to predict the prognosis of bladder cancer
Source: Cancer Med. 2021 Aug 10;10(18):6534–45. doi: 10.1002/cam4.4167 (PMC8446409; doi:10.1002/cam4.4167)
Supplement: Supplementary file 5 — Supplementary Material [file CAM4-10-6534-s003.docx]

**SUPPLEMENTARY FIGURE 1.** Survival analyses of immune-related lncRNAs.

**SUPPLEMENTARY FIGURE 2.** Correlation between the 9-lncRNAs signature and the immune cell infiltration (Figure 7A-F for the training data set and Figure 7G-I for the testing data set).
